# Critical appraisal of a non-invasive model to derive pulmonary capillary wedge pressure from cardiac magnetic resonance in heart failure patients: insights from a large Portuguese Observational Study

**DOI:** 10.1093/ehjimp/qyad017

**Published:** 2023-08-14

**Authors:** Sérgio Maltês, Mariana Sousa Paiva, Rita Reis Santos, Bruno M L Rocha, Gonçalo J L Cunha, Cláudia Silva, Sara Guerreiro, Pedro Freitas, João Abecasis, António M Ferreira

**Affiliations:** Cardiology Department, Hospital Santa Cruz, Centro Hospitalar Lisboa Ocidental, Av. Prof. Dr. Reinaldo dos Santos, 2790-134 Lisbon, Portugal; Cardiology Department, Hospital Santa Cruz, Centro Hospitalar Lisboa Ocidental, Av. Prof. Dr. Reinaldo dos Santos, 2790-134 Lisbon, Portugal; Cardiology Department, Hospital Santa Cruz, Centro Hospitalar Lisboa Ocidental, Av. Prof. Dr. Reinaldo dos Santos, 2790-134 Lisbon, Portugal; Cardiology Department, Hospital Santa Cruz, Centro Hospitalar Lisboa Ocidental, Av. Prof. Dr. Reinaldo dos Santos, 2790-134 Lisbon, Portugal; Cardiology Department, Hospital Santa Cruz, Centro Hospitalar Lisboa Ocidental, Av. Prof. Dr. Reinaldo dos Santos, 2790-134 Lisbon, Portugal; Cardiology Department, Hospital Santa Cruz, Centro Hospitalar Lisboa Ocidental, Av. Prof. Dr. Reinaldo dos Santos, 2790-134 Lisbon, Portugal; Cardiology Department, Hospital Santa Cruz, Centro Hospitalar Lisboa Ocidental, Av. Prof. Dr. Reinaldo dos Santos, 2790-134 Lisbon, Portugal; Cardiology Department, Hospital Santa Cruz, Centro Hospitalar Lisboa Ocidental, Av. Prof. Dr. Reinaldo dos Santos, 2790-134 Lisbon, Portugal; Cardiology Department, Hospital Santa Cruz, Centro Hospitalar Lisboa Ocidental, Av. Prof. Dr. Reinaldo dos Santos, 2790-134 Lisbon, Portugal; Faculdade de Ciências Médicas da Universidade Nova de Lisboa, Nova Medical School, Lisbon, Portugal; Cardiology Department, Hospital Santa Cruz, Centro Hospitalar Lisboa Ocidental, Av. Prof. Dr. Reinaldo dos Santos, 2790-134 Lisbon, Portugal

Increased left ventricular (LV) filling pressure (LVFP) is a hallmark of heart failure (HF), with significant prognostic implications.^1^ Currently, pulmonary capillary wedge pressure (PCWP), measured by right heart catheterization (RHC), remains the main metric for assessing LVFP. However, RHC is an invasive procedure, with limited availability and low but non-negligible risks. Transthoracic echocardiography has potential as an alternative, but diastolic dysfunction assessment can be challenging, relying on multiple echocardiographic parameters and operator expertise.

Cardiac magnetic resonance (CMR) is increasingly used to evaluate HF patients, but the haemodynamic information it provides is somewhat limited. Recently, Garg *et al.*^2^ proposed a physiological model to estimate PCWP from CMR data (CMR-PCWP). In a cohort of more than 800 patients referred for dyspnoea assessment [of whom only 7% had reduced or mid-range left ventricular ejection fraction (LVEF)], the authors were able to derive a model to estimate PCWP through CMR (CMR-PCWP) using two simple measurements: left ventricle mass (LVM) and left atrial volume (LAV). They found a correlation between CMR-PCWP and RHC-PCWP. Moreover, elevated CMR-PCWP had significant prognostic implications.

To determine whether those findings translate to HF with reduced ejection fraction, we conducted a retrospective cohort study of 578 consecutive patients with LVEF <50% who underwent CMR at our centre between 2015 and 2022 (mean age 63 ± 14 years, 72% male, mean LVEF 34 ± 10%, 45% ischaemic aetiology). Standard LVM and LAV measurements were used to calculate CMR-PCWP as per the proposed model. Overall, the mean CMR-PCWP was 16 ± 4 mmHg, with 298 patients (52%) showing values ≥15 mmHg. Patients with elevated CMR-PCWP were significantly older, had lower LVEF and right ventricle ejection fraction, higher ventricular volumes and higher lung water density values, a novel prognostic marker in HF.^3^ During a median follow-up of 25 (13–51) months, 69 deaths and 72 HF hospitalizations were observed. A multivariate Cox regression analysis was performed after univariate analysis to identify which variables predicted a combined endpoint of all-cause death or HF hospitalization. At multivariate analysis, CMR-PCWP proved to be an independent predictor of the combined endpoint [hazard ratio (HR) 1.067, 95% confidence interval (CI) 1.029–1.105, *P* < 0.001; adjusted for age, sex, New York Heart Association class, LVEF and NT-proBNP, supplementary data online, *Figure S1*]. However, we also found a very strong correlation between CMR-PCWP and LAV, with 95% of the variance of CMR-PCWP explained by LAV (*R*^2^ = 0.947, *Figure 1*). Furthermore, CMR-PCWP variance was only mildly determined by LVM (*R*^2^ = 0.178). While LVM showed a lower predictive power at multivariate analysis (HR 1.044 per 10 g, 95% CI 0.998–1.092, *P* = 0.061, *χ*^2^ 8094), both LAV and CMR-PCWP had a similar discriminative capability in predicting the combined endpoint (LAV—HR 1.047 per 10 mL, 95% CI 1.019–1.075, *P* = 0.001, χ^2^ 36 470; CMR-PCWP—HR 1.067, 95% CI 1.029–1.105, χ^2^ 38 951; C-statistic 0.67 vs. 0.67, *P*-value for comparison = 0.724). Moreover, the CMR-PCWP model did not improve LAV performance in predicting the combined endpoint (Net Reclassification Index 0.04, *P*-value 0.244).

**Figure 1:**
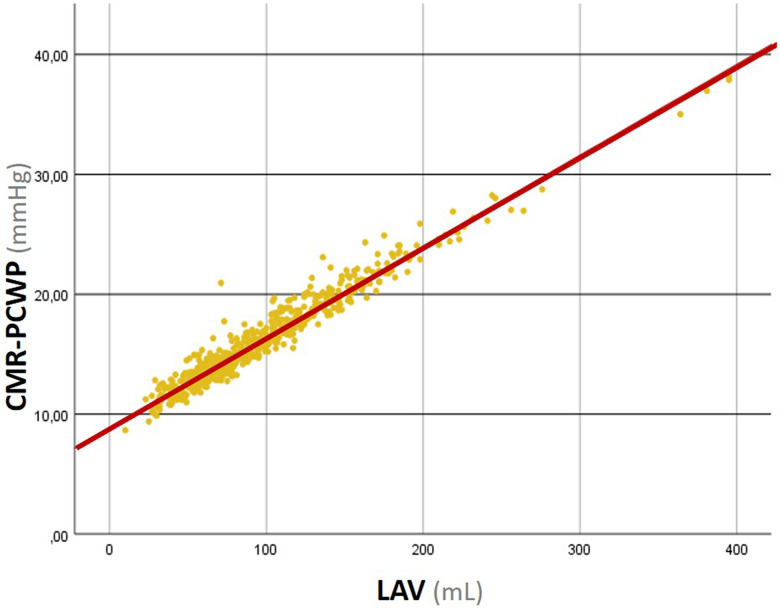
Correlation analysis between CMR-PCWP and LAV. CMR-PCWP, cardiac magnetic resonance-derived pulmonary capillary wedged pressures; LAV, left atrial volume.

Our findings suggest that in HF patients with LVEF <50%, the proposed CMR-PCWP model might be a mere surrogate of LAV. In our cohort, we were not able to find additional diagnostic or prognostic information on the CMR-PCWP model over other already established CMR measurements. However, it should be highlighted that the original purpose of the CMR-PCWP model (unexplained dyspnoea) is different from the setting where it was tested in our cohort.

Future studies must attempt to further refine the CMR-PCWP model and determine how it compares to other novel CMR-derived metrics in HF, such as pulmonary transit time and pulmonary blood volume analysis.^4,5^ As it stands, the search must continue for different CMR markers of diastolic dysfunction and increased LVFP in HF patients.

## Supplementary Material

qyad017_Supplementary_Data

## Data Availability

The data sets used in this paper are available from the corresponding author on reasonable request.

## Lead author biography

Dr Sérgio Maltês, born in March 30th 2023 in Setúbal, Portugal, is a final-year Cardiology Resident in Hospital de Santa Cruz, Centro Hospitalar de Lisboa Ocidental, a Portuguese reference centre in interventional cardiology and heart transplantation. With particular interest in clinical cardiology, echocardiography and heart failure, Dr Sérgio Maltês is an active member of the advanced heart failure and transplantation team of Hospital de Santa Cruz.
