# Supplementary figures and images for: Critical appraisal of a non-invasive model to derive pulmonary capillary wedge pressure from cardiac magnetic resonance in heart failure patients: insights from a large Portuguese Observational Study
Source: Eur Heart J Imaging Methods Pract. 2023 Aug 14;1(1):qyad017. doi: 10.1093/ehjimp/qyad017 (PMC11195800; doi:10.1093/ehjimp/qyad017)

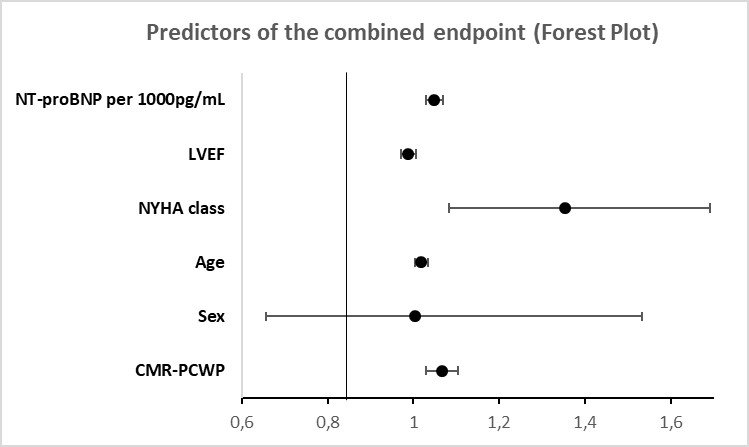

Supplement: qyad017_Supplementary_Data [file qyad017_Supplementary_Data.jpg]
